# How peer support and pressure shape career indecision: the mediating role of career calling and the buffering role of internalized prosocial values

**DOI:** 10.3389/fpsyg.2026.1914111

**Published:** 2026-08-13

**Authors:** Chenxi Li, Jiangxin He

**Affiliations:** 1 Student Affairs Department, Xi'an University of Science and Technology, Xi'an, China; 2 Student Career Development Center, Xi'an University of Science and Technology, Xi'an, China

**Keywords:** career calling, career decision-making, career indecision, collectivism, emerging adults, internalized prosocial values, moderated mediation, peer influence

## Abstract

**Introduction:**

Career indecision is a prevalent developmental challenge among emerging adults, yet the mechanisms through which peer influence relates to decisional difficulty remain poorly understood. Drawing on Social Cognitive Career Theory (SCCT) and the career calling literature, this study tested a moderated mediation model in which positive peer career support and negative peer career pressure are linked to career indecision through career calling, with internalized prosocial values moderating the association between negative peer pressure and calling.

**Methods:**

Survey data from 524 undergraduates at a comprehensive Chinese university were analyzed using confirmatory factor analysis, latent moderated structural equations (LMS), and bias-corrected bootstrap procedures.

**Results:**

Positive peer career support was positively associated with career calling (β = 0.31, *p* < 0.001), whereas negative peer career pressure was negatively associated with career calling (β = 0.27, *p* < 0.001); only negative peer pressure showed a direct association with career indecision. Career calling was negatively associated with career indecision (β = 0.46, *p* < 0.001) and fully mediated the link between positive peer support and career indecision, while partially mediating the link between negative peer pressure and career indecision. Internalized prosocial values moderated the association between negative peer pressure and career calling (interaction β = 0.18, *p* < 0.001); the conditional indirect association of negative peer pressure was substantially attenuated at high levels of value internalization.

**Discussion:**

These findings extend SCCT by showing that internalized prosocial values may serve as a contextual protective factor that attenuates the maladaptive association of peer pressure with meaning-based career development, and highlight the importance of distinguishing supportive from pressuring peer dynamics in understanding career decision-making among emerging adults in a collectivist cultural context. Given the cross-sectional design, longitudinal replication is needed to corroborate the proposed directional relationships.

## 1 Introduction

A growing proportion of young people enter higher education with unclear or fluctuating career plans (OECD, 2025). The latest OECD report on teenage career preparation estimates that around 39% of 15-year-olds in OECD countries cannot name a specific occupational expectation, and longitudinal analyses link such uncertainty to poorer employment outcomes a decade later (OECD, 2025). In China, the simultaneous expansion of higher education and the polarization of the labor market have intensified the difficulty of forming stable career goals during the undergraduate years, especially in application-oriented institutions where students often experience limited family-based career resources and underdeveloped institutional advising systems (Gao et al., 2025; Wang et al., 2026, 2023). Career indecision, defined as the difficulty in committing to a specific career direction during career decision making, has long been recognized as a developmental task that, when unresolved, is associated with poorer adjustment, lower career adaptability, and reduced well-being in emerging adulthood (Gati et al., 1996; Parola and Marcionetti, 2022; Argyropoulou et al., 2021). Importantly, the culturally collectivist orientation prevalent in Chinese society (Hofstede, 2001; Triandis, 1995) renders peer influence particularly salient during the transition to adulthood, because individuals with interdependent self-construals tend to weigh relational information more heavily in personal decisions (Singelis, 1994; Kağıtçıbaşı, 2007).

Research has shown that career indecision is shaped not only by intrapersonal factors but also by external influences, in particular parental and teacher career-related behaviors (Parola and Marcionetti, 2022; Wang et al., 2026, 2024). Yet peer-based influences have received markedly less empirical attention, even though late adolescence and early adulthood represent precisely the developmental window in which peers become the most salient socializing agents (Felsman and Blustein, 1999; Cheung and Arnold, 2014; Schellenberg et al., 2022). In Chinese universities, dormitory-based collective living, dense peer networks, and intensive group-based instruction characterize daily student life, making peer career-related behaviors a powerful yet understudied contextual force (Wang et al., 2025b; Zhou et al., 2024).

Understanding the psychological mechanism through which peer influence relates to career outcomes is equally important. The career calling literature offers a promising candidate. Calling, conceptualized as a meaning-based orientation toward work that integrates a sense of purpose, prosocial contribution, and identity coherence, has been shown to be negatively associated with career indecision by providing a stable internal compass for decision making (Duffy et al., 2018; Chen and Zhang, 2023; Zhang and Li, 2026). To date, however, the joint operation of peer influence and career calling has rarely been examined within the same model, and whether this meaning-based pathway varies across contextual conditions remains largely untested.

A potentially important contextual condition is the degree to which students have internalized prosocial values. In Chinese universities, curricula emphasizing civic responsibility, ethical reasoning, and prosocial commitment are institutionally embedded and have been linked to value identity, sense of belonging, and academic adjustment (Li et al., 2022; Zhao, 2022; Xue et al., 2024). Whether the extent of internalizing prosocial values attenuates the maladaptive association of peer pressure with career meaning, and thereby with indecision, has not yet been tested. Such a test is theoretically informative because it specifies a contextual boundary condition for the peer-to-outcome pathway, and it is practically informative because value internalization is a target that universities can actively promote (Tong, 2025; Tian, 2025). Recent international work further suggests that perceived environmental and contextual barriers are increasingly central to understanding career development among young adults in uncertain times, including among students who rely most heavily on peer reference groups (Sheu et al., 2018; Duffy et al., 2016).

The present study contributes to the literature in several ways. Conceptually, by separating positive peer career support from negative peer career pressure, the study moves beyond an undifferentiated treatment of peer influence and addresses its directionality. Mechanistically, by positioning career calling as a meaning-based mediator, the study tests a pathway that complements the more frequently studied cognitive-efficacy mechanism (Liu et al., 2023b; Xu et al., 2025) and aligns with contemporary career construction perspectives (Savickas, 2013; Rudolph et al., 2017). Contextually, by introducing internalized prosocial values as the moderator on the pressure-to-calling path, the study identifies a targetable psychological resource that may attenuate the maladaptive associations of negative peer dynamics, and it situates these processes within the collectivist cultural context that amplifies peer salience (Hofstede, 2001; Triandis, 1995). The remainder of the paper develops the theoretical framework and hypotheses (Section 2), describes the methodology (Section 3), reports the results (Section 4), and discusses implications and limitations (Sections 5–6).

## 2 Literature review and hypothesis development

### 2.1 Theoretical foundation and framework

The conceptual model of the present study is grounded in the Social Cognitive Career Theory (SCCT) of Lent, Brown, and Hackett (Lent et al., 1994; Lent and Brown, 2019), which posits that career-related cognitions, behaviors, and outcomes are jointly shaped by the interaction of personal attributes, environmental influences, and learning experiences. Within SCCT, contextual supports and barriers are not merely background variables but active inputs into the cognitive appraisals through which individuals form goals and persist in their career-related actions (Lent and Brown, 2019; Sheu et al., 2018). SCCT specifies that contextual affordances operate on career outcomes through two cognitive-person variables: self-efficacy expectations and outcome expectations (Lent et al., 1994). The present model extends this specification by arguing that career calling constitutes an additional meaning-based cognitive-person variable through which contextual peer influences relate to career indecision. We prefer calling over the more frequently studied self-efficacy pathway because, whereas self-efficacy captures confidence in the ability to perform career-related tasks, calling captures the sense of purpose and direction that organizes career-related decisions (Duffy et al., 2018; Dik and Duffy, 2009). In contexts where information about career options is abundant but coherence and personal meaning are scarce, calling may be a more proximal cognitive-person variable linking peer experiences to decisional clarity (Hirschi and Pang, 2023; Praskova et al., 2015). This extension is consistent with recent integrative proposals that broaden SCCT's cognitive-person layer beyond self-efficacy and outcome expectations to include meaning-based constructs (Hirschi and Koen, 2021; Akkermans and Hirschi, 2023).

Peer behaviors occupy a distinctive position in this contextual layer: peers are sufficiently similar to be sources of vicarious learning and social comparison, and sufficiently embedded in the daily life of the student to provide repeated exposure across academic and non-academic settings (Felsman and Blustein, 1999; Cheung and Arnold, 2014; Schellenberg et al., 2022). This salience is amplified in collectivist cultural contexts. Hofstede's cultural dimensions framework identifies Chinese society as high on collectivism and power distance (Hofstede, 2001), and Triandis's distinction between horizontal and vertical collectivism suggests that peer-level social comparison in such contexts carries strong normative weight (Triandis, 1995). Singelis's construct of interdependent self-construal (Singelis, 1994), which is more prevalent in collectivist cultures, implies that individuals define themselves partly through relational harmony and in-group membership, making peer evaluation a particularly potent input into self-relevant career cognitions. Kağıtçıbaşı's model of family and human development further underscores that the autonomy-relatedness dialectic in collectivist societies shapes how young adults negotiate personal career aspirations against group expectations (Kağıtçıbaşı, 2007). Together, these cultural-psychological frameworks suggest that peer career support and peer career pressure should have stronger associations with career-related meaning in the Chinese context than undifferentiated, culturally decontextualised models would predict. SCCT therefore offers a natural lens for examining how peer-related supports and barriers are associated with career outcomes.

A complementary lens is provided by the career construction perspective, which emphasizes that career development is a meaning-making process in which individuals integrate external experiences into coherent identity narratives (Savickas, 2013; Hirschi and Koen, 2021). From this standpoint, the association of peer influence with career outcomes is unlikely to be unmediated; rather, it is filtered through psychological constructs that organize meaning and direction. Career calling is one of the central such constructs. Defined as a transcendent summons or sense of purpose that leads individuals to approach their work as a contribution to a greater good, calling has been shown to be associated with career commitment, adaptability, and well-being across diverse samples (Duffy et al., 2018; Dik and Duffy, 2009; Praskova et al., 2015; Beloborodova and Leontiev, 2024).

**Figure 1:**
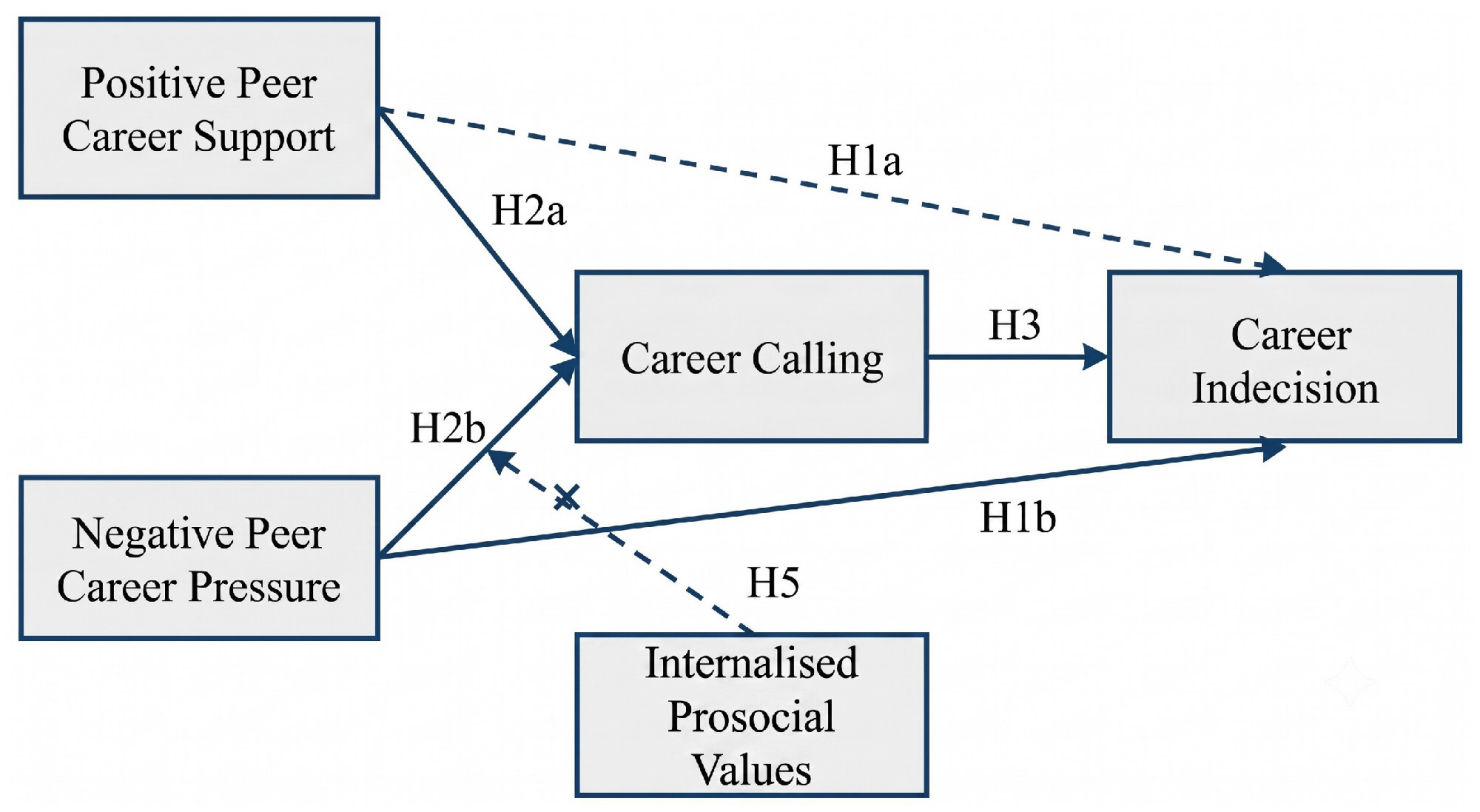
Conceptual framework of the moderated mediation model. Positive peer career support and negative peer career pressure are linked to career indecision through career calling. Internalized prosocial values moderate the path from negative peer pressure to career calling.

Bringing these strands together yields the conceptual model summarized in Figure 1. Peer influence is treated as a two-dimensional contextual input. The positive dimension captures peer career-related support, namely the extent to which peers provide informational, emotional, and instrumental assistance for career planning. The negative dimension captures peer career-related pressure, namely the extent to which peers generate social comparison stress, expectations of conformity, and competitive anxiety (Xu and Li, 2024; Wen and Jin, 2025). Career calling is positioned as the meaning-based mediator linking peer influence to career indecision. Internalized prosocial values, defined as the degree to which civic, ethical, and prosocial commitments have been personally integrated into the student's value system (Ryan and Deci, 2017; Tong, 2025), are positioned as the moderator of the pressure-to-calling pathway, on the theoretical expectation that deeply held prosocial commitments shield meaning-based career development from the negative associations of peer comparison. This construct is conceptually distinct from mere exposure to values-education curricula; it captures an individual psychological resource that, once internalized, provides a stable, non-peer-referenced source of meaning (Vansteenkiste et al., 2020; Zhang and Fagan, 2016).

### 2.2 Peer influence and career indecision

Career indecision refers to the difficulty experienced by individuals when they are required to commit to a career choice, and it is most usefully conceptualized as a multidimensional construct encompassing lack of information, lack of readiness, and inconsistent information (Gati et al., 1996). In Chinese undergraduate samples, career indecision has been consistently associated with diminished career adaptability, lower career decision-making self-efficacy, perfectionistic concerns, and reduced subjective well-being (Wang et al., 2026; Xu et al., 2025; Liu et al., 2023b; Chen et al., 2022). Recent person-centered work in European samples has further shown that career indecision presents as a constellation of distinct profiles rather than a single difficulty type, supporting a heterogeneous view of the construct (Parola and Marcionetti, 2024). While much of the research on antecedents of career indecision has focused on parental and teacher behaviors (Parola and Marcionetti, 2022; Wang et al., 2024), peers play an equally consequential role during the university years because peer groups in Chinese universities are spatially and temporally dense; dormitory cohabitation, class-based small groups, and the collective rhythms of university life ensure that peers are an everyday presence in students' decision processes (Cheung and Arnold, 2014; Wang et al., 2025b). Moreover, peers serve as a natural reference group for social comparison about career options, internship choices, and labor market signals, which makes them particularly relevant to the formation or destabilization of career direction (Xu and Li, 2024; Wen and Jin, 2025). Longitudinal evidence from Chinese samples further indicates that social support is associated with improved career adaptability primarily by enhancing career decision-making self-efficacy, underscoring the developmental relevance of relational inputs during the university years (Hou et al., 2019).

The empirical literature suggests that peer influence operates through both supportive and pressuring pathways, and that conflating these two faces of peer influence may obscure their distinct associations (Felsman and Blustein, 1999; Marcionetti and Zammitti, 2025; Aydemir Dev and Bayram Arlı, 2025). Positive peer career support, which includes peers sharing career information, modeling exploratory behavior, and providing emotional encouragement, is theorized to reduce uncertainty and to enrich the informational base from which decisions are made (Schellenberg et al., 2022; Zhou et al., 2024; Wang, 2025). Negative peer career pressure, in contrast, which includes upward social comparison, conformity expectations, and peer-driven competitive anxiety, is theorized to amplify uncertainty by introducing externally referenced standards that are difficult to reconcile with one's own emerging preferences (Shang and Bao, 2025; Wen and Jin, 2025). In collectivist cultural contexts, where maintaining group harmony and meeting in-group expectations are valued, the pressuring dimension of peer influence may carry particular weight (Triandis, 1995; Singelis, 1994). Recent work on contemporary career orientations underscores that such bivalent peer dynamics are increasingly central to the developmental ecology of young adults (Hirschi and Koen, 2021; Akkermans and Hirschi, 2023). We therefore propose:

**H1a**. Positive peer career support is negatively associated with career indecision among Chinese university students.

**H1b**. Negative peer career pressure is positively associated with career indecision among Chinese university students.

### 2.3 Career calling as a meaning-based mediator

Career calling has emerged as one of the most influential constructs in contemporary career psychology. Duffy and colleagues define calling as a transcendent summons that leads individuals to approach their work with a sense of purpose and prosocial orientation (Duffy et al., 2018; Dik and Duffy, 2009; Duffy et al., 2016). Calling differs from generic career interest in that it integrates personal meaning, identity, and a perceived obligation to contribute, and it differs from career self-efficacy in that it concerns the *why* of work rather than the *can* of work (Praskova et al., 2015; Hirschi and Pang, 2023). Empirical research has linked higher calling to greater career commitment, more proactive career behavior, and lower career indecision (Zhang et al., 2017, 2022; Chen et al., 2024). Among Chinese undergraduates specifically, calling has been associated with stronger vocational identity, more sustained career commitment, and higher life satisfaction (Zhang et al., 2015; Jia et al., 2021; Parola et al., 2023). The role of prosocial modeling by salient adults and faculty in strengthening this meaning-based commitment has been particularly visible in recent work on Chinese medical students, where exposure to prosocial role models was associated with stronger career commitment (Wang et al., 2022).

The logic of treating calling as a mediator of peer influence has two pillars. On the one hand, peer-related experiences are sources of vicarious learning and social comparison that shape the meaning students attach to their future work. When peers model thoughtful career exploration and share authentic narratives about their developing vocational identities, students are more likely to integrate work into a coherent personal mission (Schellenberg et al., 2022; Felaco et al., 2023). Conversely, when peer interactions are dominated by competitive pressure and externally referenced standards, students are pushed toward extrinsic, status-driven framings of work that crowd out meaning-based motivation (Hirschi and Koen, 2021; Xu and Li, 2024; Wen and Jin, 2025). On the other hand, the meaning-based literature is consistent with self-determination theory (Ryan and Deci, 2017; Vansteenkiste et al., 2020), which suggests that authentic, internally integrated motivations are sustained by relatedness experiences that affirm autonomy and competence, and are eroded by relatedness experiences that introduce conditional regard. Together, these arguments lead to the following hypotheses:

**H2a**. Positive peer career support is positively associated with career calling.

**H2b**. Negative peer career pressure is negatively associated with career calling.

**H3**. Career calling is negatively associated with career indecision.

**H4a**. Career calling mediates the association between positive peer career support and career indecision.

**H4b**. Career calling mediates the association between negative peer career pressure and career indecision.

### 2.4 Internalized prosocial values as a buffering moderator

In Chinese higher education, values education, often referred to as ideological and political education, is delivered through a comprehensive institutionally embedded curriculum that emphasizes civic responsibility, ethical reasoning, and prosocial commitment (Tong, 2025; Zhang and Fagan, 2016; Tian, 2025). This curriculum has, in recent years, expanded beyond rote political theory to engage students with moral reasoning, social responsibility, and the integration of personal values with civic life (Li et al., 2022; Zhao, 2022; Xue et al., 2024). Although the specific content of values education varies across national contexts, the broader phenomenon of institutionally embedded character or civic education is recognizable across many higher education systems.

Critically, the moderator in the present model is not exposure to values-education curricula per se, but rather the degree to which students have internalized the prosocial and civic values that such curricula promote. This distinction is important because curriculum exposure is an institutional input, whereas internalized prosocial values represent an individual psychological resource that results from the integration of externally presented values into the self-system (Ryan and Deci, 2017; Vansteenkiste et al., 2020). Self-determination theory distinguishes several stages of value regulation, from external compliance to identified and integrated regulation (Ryan and Deci, 2017). Only when values are integrated, that is, when students experience prosocial commitments as personally meaningful and volitional, do these commitments function as stable anchors for autonomous motivation (Vansteenkiste et al., 2020; Hirschi, 2020). The construct of internalized prosocial values, as used here, therefore captures this integrated stage of value regulation.

Internalized prosocial values should attenuate the negative association between peer pressure and career calling because they provide a non-peer-referenced source of meaning. When students have deeply integrated civic and ethical commitments, they possess a non-peer-referenced source of meaning from which to draw, which weakens the capacity of upward social comparison and conformity pressure to erode their sense of calling. This argument is consistent with self-determination theory's account of how internalized values supports autonomous motivation under contextual challenge (Ryan and Deci, 2017; Hirschi, 2020; Vansteenkiste et al., 2020), with recent Chinese-context evidence that values-education quality is positively associated with value identity, social responsibility, and psychological adjustment (Zhao, 2022; Xue et al., 2024; Tong, 2025), and with recent evidence that internalized values are associated with clearer career choices through enhanced career adaptability among Chinese engineering undergraduates (Wang et al., 2025a). It is also consistent with the broader career construction view that prosocial career strivings are protective against the negative associations of purely extrinsic comparison (Hirschi and Pang, 2023).

It is important to note that internalized prosocial values are conceptually distinct from career calling, even though both constructs involve meaning and purpose. Career calling refers to a domain-specific orientation toward one's future work that integrates a sense of transcendent summons, purposeful work, and prosocial contribution (Duffy et al., 2018). Internalized prosocial values, by contrast, refer to a domain-general integration of civic and ethical commitments into the self-system that is not restricted to the career domain (Ryan and Deci, 2017). Values provide the motivational substrate from which calling may emerge, but they are neither sufficient for calling nor identical to it. This conceptual distinction is tested empirically through discriminant validity analysis (Section 4.2).

We do not expect internalized prosocial values to moderate the positive peer-to-calling path symmetrically, because positive peer support and prosocial values are likely to operate through overlapping integration mechanisms, leading to additive rather than interactive associations (Wang et al., 2025b; Wang, 2025). We therefore propose:

**H5**. Internalized prosocial values moderate the negative association between peer career pressure and career calling, such that the association is attenuated at higher levels of prosocial value internalization.

## 3 Methodology

### 3.1 Participants and procedure

Following a brief pilot study (*n* = 128) used to refine the internalized prosocial values items and the peer career influence subscales, formal data collection was conducted between October 2025 and January 2026 at a comprehensive university in northwestern China. The institution was deliberately selected because its broad disciplinary coverage and balanced student composition support reasonable generalizability to comprehensive universities in China (Wang et al., 2023; Gao et al., 2025), although results from a single institution should be interpreted with caution and replication across institutional types is needed. With the cooperation of the Student Affairs Department and class counselors, an online survey link was distributed to undergraduates across all four-year cohorts. Stratification was conducted proportionally on year of study, gender, and disciplinary group (STEM vs. non-STEM) to align the sample with the institutional enrolment profile. Participation was voluntary and anonymous; students who agreed to a digital informed consent statement were directed to the survey. Of 587 returned questionnaires, 63 were excluded as follows: 24 for incomplete responses (more than 10% missing items), 21 for failure on two embedded attention-check items (e.g., “Please select ‘Agree' for this item”), and 18 for implausibly fast completion times (below the two-minute threshold determined by pilot-study response-time distributions). The final analytic sample comprised *N* = 524.

The sample composition is summarized in Table 1. The age range was 18 to 24 years (*M* = 20.41, *SD* = 1.36). The gender distribution was 287 male (54.8%) and 237 female (45.2%), reflecting the institution's slight male majority. Year of study was approximately balanced across the four undergraduate cohorts. Discipline was coded as STEM (62.2%) vs. non-STEM (37.8%), and 41.4% of participants identified as first-generation university students. Rural and urban origin were almost evenly distributed, mirroring the institution's recruitment profile.

**Table 1:** Sample composition (*N* = 524).

Characteristic	*n*	%
Gender, male	287	54.8
Gender, female	237	45.2
Year 1	132	25.2
Year 2	145	27.7
Year 3	138	26.3
Year 4	109	20.8
Major, STEM	326	62.2
Major, non-STEM	198	37.8
First generation	217	41.4
Continuing generation	307	58.6
Origin, rural	263	50.2
Origin, urban	261	49.8

The survey protocol and instruments were approved by the institutional ethics review board prior to data collection. All participants were informed of the study's purpose, the voluntary and anonymous nature of participation, the right to withdraw at any time without consequence, and the aggregate use of data. To reduce common method bias, the order of scales was randomized across participants, and the internalized prosocial values scale was separated from the career-related scales by a battery of demographic and filler items (Podsakoff et al., 2003).

### 3.2 Measures

All scales were administered in Mandarin Chinese. Established English instruments were translated into Chinese using forward and backward translation by two bilingual researchers, with discrepancies resolved through expert discussion. All items used a five-point Likert scale ranging from 1 (*strongly disagree*) to 5 (*strongly agree*) unless otherwise indicated.

#### 3.2.1 Peer career influence

Peer career influence was measured by an eight-item scale adapted from the peer career support literature (Felsman and Blustein, 1999; Cheung and Arnold, 2014; Schellenberg et al., 2022) and developed further to capture the bidimensional positive and negative dynamics emphasized in the present model. The Positive Peer Career Support sub-scale (four items) captured the informational, emotional, and instrumental support that peers provided for career-related decisions (example item: “My peers share useful information about career options with me”). The Negative Peer Career Pressure sub-scale (four items) captured competitive social comparison, conformity expectations, and peer-driven anxiety (example item: “I feel pressured to choose a career because of what my peers are doing”). The bidimensional structure was supported by exploratory factor analysis in the pilot sample (*n* = 128; two-factor solution explaining 68.4% of variance; all primary loadings >0.65; no cross-loadings >0.30) and confirmed in the main sample.

#### 3.2.2 Career calling

Career calling was measured by a twelve-item adaptation of the Calling and Vocation Questionnaire developed by Dik, Eldridge, Steger, and Duffy (Dik et al., 2012), which captures the transcendent summons, the purposeful work orientation, and the prosocial contribution dimensions of calling. The conceptual basis of the instrument follows the calling framework articulated by Duffy and colleagues (Duffy et al., 2018; Dik and Duffy, 2009). Sample items include “I believe I have been called to my future career,” “My future work will help me find meaning in life,” and “I want to use my career to contribute to the common good.” The Chinese adaptation has demonstrated satisfactory psychometric properties in prior research with undergraduate samples (Zhang et al., 2015; Jia et al., 2021).

#### 3.2.3 Internalized prosocial values

A six-item scale measuring internalized prosocial values was developed for this study, drawing on the literature on value internalization within self-determination theory (Ryan and Deci, 2017; Vansteenkiste et al., 2020) and on recent Chinese-context research on values-education engagement (Tong, 2025; Xue et al., 2024; Li et al., 2022). The scale was designed to capture the degree to which students have personally integrated prosocial and civic commitments into their value system, rather than to assess mere exposure to or satisfaction with values-education curricula. Scale development followed a multi-step procedure. An initial pool of twelve items was generated on the basis of the value-internalization and prosocial-values literatures, reviewed by a panel of three experts in career psychology, civic education, and psychometrics, and piloted with the pilot sample (*n* = 128). Based on expert review and pilot-study item-total correlations (all retained items >0.55), six items were selected. Exploratory factor analysis in the pilot sample produced a clear unidimensional solution (single eigenvalue >1; 62.8% of variance explained; all factor loadings >0.70). The six items are: (a) “Civic responsibility is a core part of who I am”; (b) “I have genuinely integrated the ethical principles I learned into my daily behavior”; (c) “Contributing to society is personally meaningful to me, not just something I am told to do”; (d) “The prosocial values I hold guide my important life decisions”; (e) “I feel a deep personal commitment to acting ethically in my future career”; (f) “When I reflect on my values, civic and ethical commitments come readily to mind.” The items were designed to tap the identified and integrated stages of value regulation as defined by self-determination theory (Ryan and Deci, 2017), distinguishing the construct from external compliance or introjected regulation. Importantly, none of the items references the institutional curriculum or teaching quality, ensuring that the construct captures an individual psychological resource rather than an institutional exposure variable. The unidimensional structure was confirmed in the main sample by a single-construct CFA [χ^2^(9) = 22.67, *p* = 0.007, CFI = 0.987, TLI = 0.978, RMSEA = 0.054, SRMR = 0.023; standardized factor loadings ranged from 0.72 to 0.85]. Cronbach's alpha was 0.88 and composite reliability was 0.90. To establish nomological validity, internalized prosocial values correlated positively with an established four-item prosocial motivation sub-scale from the Revised Prosocial Motivation Scale (Grant, 2008) administered to a sub-sample (*n* = 196, *r* = 0.58, *p* < 0.001), supporting convergent validity with a conceptually related but independently measured construct. Discriminant validity between internalized prosocial values and career calling is reported in Section 4.2.

#### 3.2.4 Career indecision

Career indecision was measured by a twelve-item adaptation of the short form of the Career Decision Difficulties Questionnaire (Gati et al., 1996), capturing the three families of difficulty (lack of readiness, lack of information, and inconsistent information). Sample items include “I find it difficult to make a career decision because I don't know how to make this kind of decision” and “I find it difficult to make a career decision because I am still uncertain about my career preferences.” The Chinese adaptation has been used in recent Chinese-context studies with satisfactory reliability (Wang et al., 2026; Xu et al., 2025; Liu et al., 2023b).

#### 3.2.5 Control variables

To reduce confounding, gender (male = 0, female = 1), year of study (1 to 4), disciplinary group (STEM = 0, non-STEM = 1), first-generation status (0/1), and rural origin (0/1) were included as control variables in the structural model.

### 3.3 Data analysis strategy

The analytic procedure followed four stages. The measurement model was evaluated by confirmatory factor analysis (CFA) using maximum likelihood estimation with robust standard errors. Model fit was assessed via the comparative fit index (CFI), the Tucker–Lewis index (TLI), the root mean square error of approximation (RMSEA), and the standardized root mean square residual (SRMR), with conventional thresholds of CFI and TLI ≥0.90, RMSEA ≤ 0.08, and SRMR ≤ 0.08 (Hu and Bentler, 1999; Kline, 2023). Composite reliability and average variance extracted were computed per construct, and discriminant validity was assessed by the Fornell–Larcker criterion (Fornell and Larcker, 1981). Given that students were nested within dormitory groups, intraclass correlation coefficients (ICCs) were computed for all study variables to determine whether multilevel modeling was warranted (Hox, 2010).

The structural model was estimated with the same robust ML procedure. Standardized path coefficients, 95% confidence intervals, and overall model fit were reported. Common method bias was assessed via Harman's single-factor test, a common latent factor (CLF) test in which a single latent factor was added to all measured indicators in the CFA (Podsakoff et al., 2003; Marsh et al., 2004), and a theoretically unrelated marker variable (a four-item aesthetic preference scale unrelated to career constructs).

The moderated mediation model was estimated entirely within the latent-variable framework using the Latent Moderated Structural Equations (LMS) approach implemented in Mplus 8.10 (Klein and Moosbrugger, 2000; Maslowsky et al., 2015). The LMS approach creates the latent interaction term (negative peer pressure × internalized prosocial values) through numerical integration rather than product indicators, which avoids the non-normality and model-identification problems that arise when product-indicator methods are used, and it retains full control for measurement error in both the main-effect and interaction terms (Klein and Moosbrugger, 2000). Because the LMS estimator does not produce conventional chi-square-based fit indices, model fit was assessed in two steps: the baseline structural model without the interaction term was evaluated using standard robust ML fit indices, and the model including the latent interaction was compared to the baseline model using the log-likelihood ratio test (Maslowsky et al., 2015). Conditional indirect effects were probed at the mean and at one standard deviation below and above the mean of internalized prosocial values using a bias-corrected bootstrap procedure with 5,000 resamples, and the index of moderated mediation was computed following the procedure described by Hayes (Preacher and Hayes, 2008; Hayes, 2018) and adapted for the latent-variable framework. All analyses were conducted in Mplus 8.10. As an additional analytic step, theoretically plausible alternative structural models were tested to evaluate the robustness of the proposed directional specification (Section 4.6).

## 4 Results

### 4.1 Common method bias, nesting effects, and descriptive statistics

Harman's single-factor test produced a largest single factor accounting for 28.4% of the variance, well below the conservative 50% threshold (Podsakoff et al., 2003). The CLF test showed that adding a common latent factor to the five-factor CFA did not significantly improve model fit [Δχ^2^(38) = 46.21, *p* = 0.168], and the average change in standardized factor loadings after partialling out the CLF was 0.013, indicating negligible common method variance (Marsh et al., 2004). The marker variable (aesthetic preference) showed a mean correlation of 0.04 with the main study variables, and adjusting for this shared variance did not alter any substantive path by more than 0.02, further supporting the conclusion that common method bias does not substantively threaten the findings.

Intraclass correlation coefficients (ICCs) were computed using dormitory group as the clustering variable (*k* = 137 dormitory groups, average group size = 3.82). ICCs ranged from 0.02 (career indecision) to 0.04 (negative peer career pressure), all below the conventional threshold of 0.05 recommended for consideration of multilevel modeling (Hox, 2010). Design effects (calculated as 1+[average group size − 1] × ICC) ranged from 1.06 to 1.11, well below the threshold of 2.0 at which single-level estimates become meaningfully biased. Single-level structural equation modeling was therefore judged appropriate for these data.

Means, standard deviations, and bivariate correlations are reported in Table 2. Positive peer career support was positively correlated with career calling (*r* = 0.41, *p* < 0.001) and internalized prosocial values (*r* = 0.29, *p* < 0.001), and negatively correlated with career indecision (*r* = −0.28, *p* < 0.001). Negative peer career pressure was negatively correlated with career calling (*r* = −0.36, *p* < 0.001) and positively correlated with career indecision (*r* = 0.39, *p* < 0.001). Career calling and career indecision were strongly negatively correlated (*r* = −0.55, *p* < 0.001). Internalized prosocial values were positively correlated with career calling (*r* = 0.34, *p* < 0.001) and negatively correlated with career indecision (*r* = −0.24, *p* < 0.001). The magnitudes of these correlations are consistent with prior Chinese-context studies on calling and decision-making outcomes (Jia et al., 2021; Chen and Zhang, 2023; Xu et al., 2025).

**Table 2:** Descriptive statistics, correlations, and discriminant validity.

Variable	*M*	*SD*	1	2	3	4	5
1. Positive peer career support	3.67	0.82	**0.79**				
2. Negative peer career pressure	3.12	0.91	−0.18^***^	**0.77**			
3. Career calling	3.74	0.86	0.41^***^	−0.36^***^	**0.84**		
4. Internalized prosocial values	3.55	0.87	0.29^***^	−0.12^**^	0.34^***^	**0.81**	
5. Career indecision	2.78	0.83	−0.28^***^	0.39^***^	−0.55^***^	−0.24^***^	**0.82**

*N* = 524.

^**^p < 0.01;

^***^p < 0.001.

Bold diagonal entries are $\sqrt{\text{AVE}}$.

### 4.2 Measurement model

The five-factor measurement model demonstrated acceptable fit to the data, χ^2^(550) = 1, 284.71, *p* < 0.001, χ^2^/*df* = 2.34, CFI = 0.943, TLI = 0.938, RMSEA = 0.050 (90% CI [0.046, 0.054]), SRMR = 0.041. Comparison with alternative models (a single-factor model, a three-factor model collapsing positive and negative peer pressure, and a four-factor model collapsing calling and internalized prosocial values) showed that the hypothesized five-factor model fit substantially better, supporting the distinctiveness of the constructs (Kline, 2023). A further alternative four-factor model that collapsed career calling and internalized prosocial values into a single latent factor yielded significantly worse fit [Δχ^2^(4) = 187.34, *p* < 0.001, CFI = 0.891, TLI = 0.882, RMSEA = 0.071], confirming that the two meaning-related constructs are empirically distinguishable. The heterotrait-monotrait (HTMT) ratio of correlations between career calling and internalized prosocial values was 0.38, well below the conservative 0.85 threshold (Henseler et al., 2015), providing additional discriminant validity evidence for these two constructs.

Reliability and convergent validity indices are reported in Table 3. Cronbach's alpha values ranged from 0.85 to 0.94, and composite reliability (CR) values ranged from 0.88 to 0.95, all comfortably exceeding the 0.70 threshold. Average variance extracted (AVE) values ranged from 0.59 to 0.71, all exceeding the 0.50 threshold. Discriminant validity was supported because the square root of each construct's AVE (Table 2, diagonal) exceeded its correlations with all other constructs (Fornell and Larcker, 1981).

**Table 3:** Confirmatory factor analysis: reliability and convergent validity.

Construct	Cronbach's α	CR	AVE
Positive peer career support	0.89	0.91	0.62
Negative peer career pressure	0.85	0.88	0.59
Career calling	0.92	0.94	0.71
Internalized prosocial values	0.88	0.90	0.65
Career indecision	0.94	0.95	0.68

### 4.3 Structural path analysis

**Figure 2:**
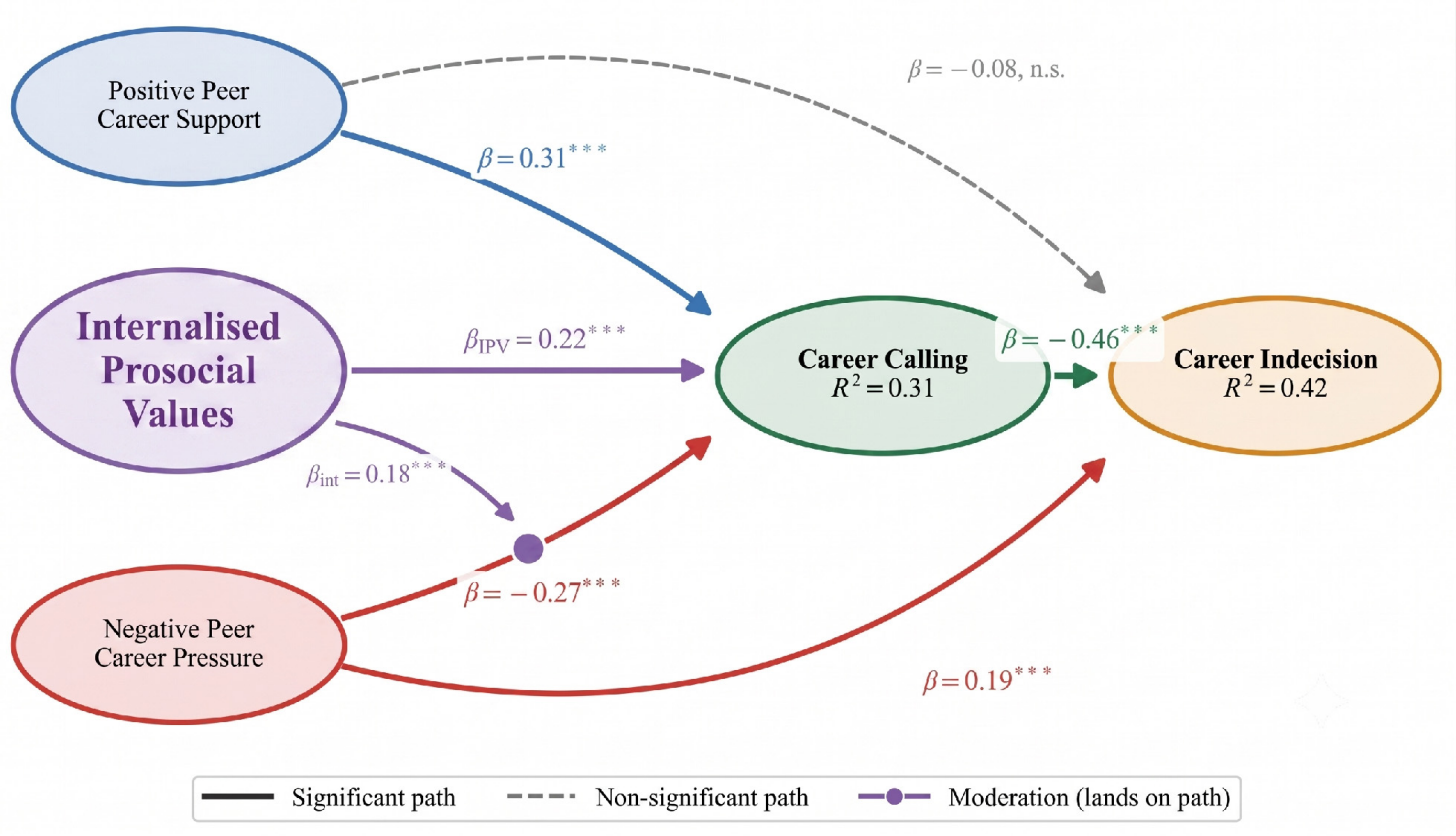
Standardized structural model solution (*N* = 524). Path coefficients are from the latent moderated structural equations (LMS) model. Solid lines indicate significant paths (*p* < 0.05) and dashed lines indicate non-significant paths. Control variables were included but are omitted for visual clarity.

The baseline structural model (without the latent interaction term) fit the data well: χ^2^(562) = 1, 311.45, *p* < 0.001, χ^2^/*df* = 2.33, CFI = 0.941, TLI = 0.937, RMSEA = 0.050, SRMR = 0.046. Adding the latent interaction term via LMS significantly improved model fit (log-likelihood difference = 12.84, Δ*df* = 1, *p* < 0.001), supporting the inclusion of the moderation term (Maslowsky et al., 2015). Standardized path coefficients are reported in Table 4 and visualized in Figure 2.

**Table 4:** Standardized path coefficients in the structural model.

Path	β	SE	95% CI	*p*
H1a: Positive peer support → Career indecision	−0.08	0.05	[−0.17, 0.02]	0.118
H1b: Negative peer pressure → Career indecision	0.19	0.04	[0.11, 0.27]	< 0.001
H2a: Positive peer support → Career calling	0.31	0.04	[0.23, 0.39]	< 0.001
H2b: Negative peer pressure → Career calling	−0.27	0.04	[−0.35, −0.18]	< 0.001
H3: Career calling → Career indecision	−0.46	0.04	[−0.54, −0.38]	< 0.001
Internalized prosocial values → Career calling	0.22	0.04	[0.13, 0.30]	< 0.001
Neg. peer pressure × Prosocial values → Calling	0.18	0.04	[0.10, 0.26]	< 0.001
Pos. peer support × Prosocial values → Calling	0.06	0.04	[−0.02, 0.14]	0.151

*N* = 524. Standardized coefficients. LMS estimation. Controls included but omitted.

The positive sign on the interaction term (negative peer pressure × internalized prosocial values, β = 0.18) combined with the negative main-effect coefficient of negative peer pressure on career calling (β = −0.27) indicates a buffering pattern: as internalized prosocial values increase, the conditional slope of negative peer pressure on calling moves toward zero.

Consistent with H1b, negative peer career pressure was positively associated with career indecision (β = 0.19, *p* < 0.001). H1a was not supported in the direct path: positive peer career support did not directly show a significant association with career indecision (β = −0.08, *p* = 0.118), although it operated indirectly via calling (Section 4.4). H2a, H2b, and H3 were all supported. As predicted by H5, the interaction between negative peer pressure and internalized prosocial values on career calling was significant (β = 0.18, *p* < 0.001); together with the negative main coefficient of negative peer pressure on calling (β = −0.27), the positive interaction indicates that the conditional slope of pressure on calling becomes less negative as prosocial value internalization rises, confirming the buffering pattern hypothesized in H5. By contrast, the parallel interaction with positive peer support was not significant (β = 0.06, *p* = 0.151), supporting the asymmetric moderation argument.

### 4.4 Mediation analysis

**Figure 3:**
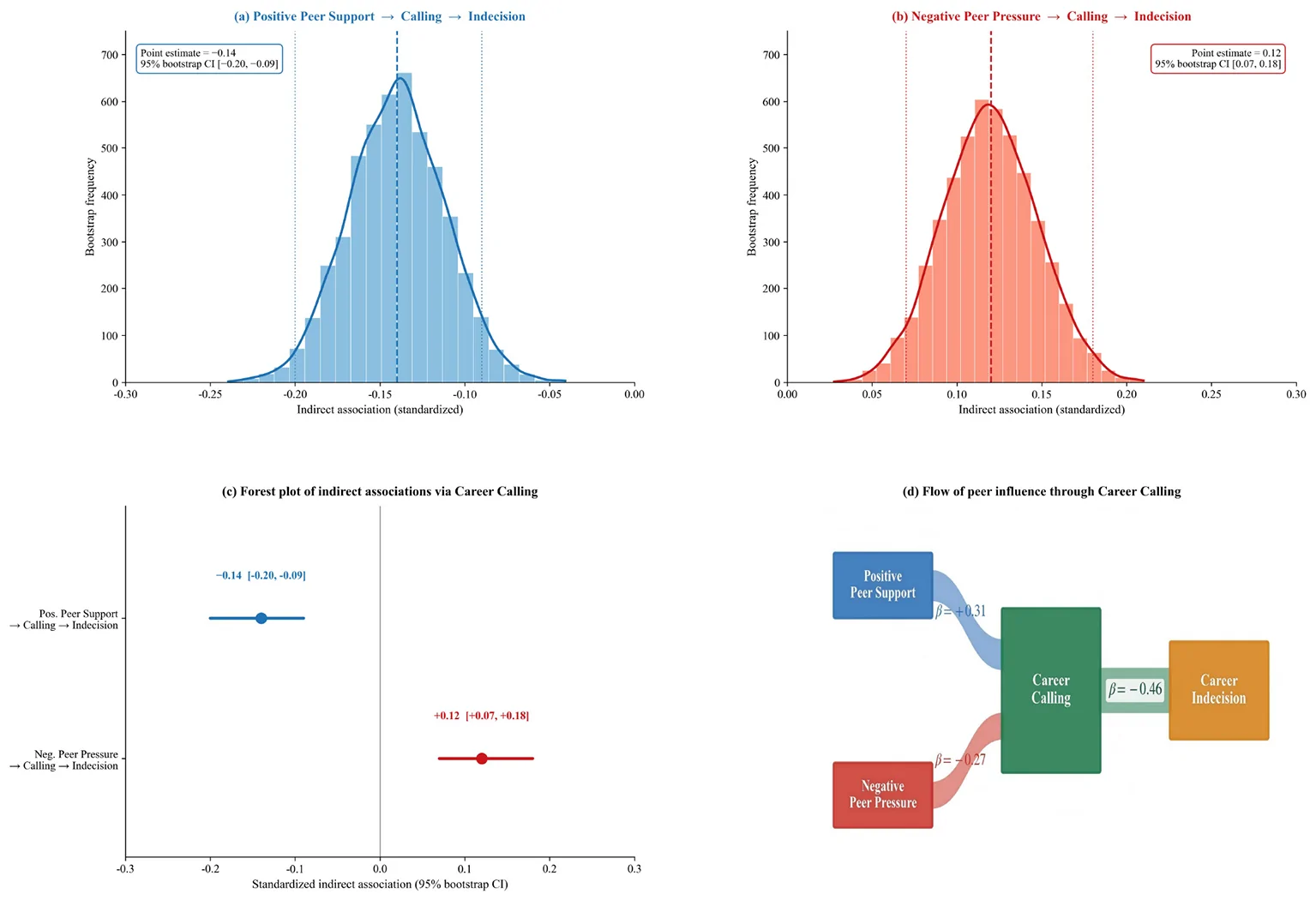
Bootstrap distributions of indirect associations via career calling (5,000 resamples).

The bias-corrected bootstrap procedure with 5,000 resamples produced the indirect associations summarized in Table 5. Career calling fully mediated the association of positive peer career support with career indecision (indirect association = −0.14, 95% CI [−0.20, −0.09]; direct path non-significant), and partially mediated the association of negative peer career pressure with career indecision (indirect association = 0.12, 95% CI [0.07, 0.18]; direct path significant). The two indirect associations differed in sign as expected and were both statistically distinct from zero, supporting H4a and H4b. The bootstrap distributions of the two indirect associations are displayed in Figure 3.

**Table 5:** Mediation analysis: indirect, direct, and total associations with career indecision.

Path	Estimate	SE	95% bootstrap CI	Proportion mediated
Positive peer career support → Career calling → Career indecision				
Indirect via calling	−0.14	0.03	[−0.20, −0.09]	63.6%
Direct	−0.08	0.05	[−0.17, 0.02]	n.a.
Total	−0.22	0.05	[−0.31, −0.13]	n.a.
Negative peer career pressure → Career calling → Career indecision				
Indirect via calling	0.12	0.03	[0.07, 0.18]	38.7%
Direct	0.19	0.04	[0.11, 0.27]	n.a.
Total	0.31	0.05	[0.22, 0.40]	n.a.

*N* = 524. Bootstrap 5,000 resamples. Standardized estimates.

### 4.5 Moderated mediation analysis

**Figure 4:**
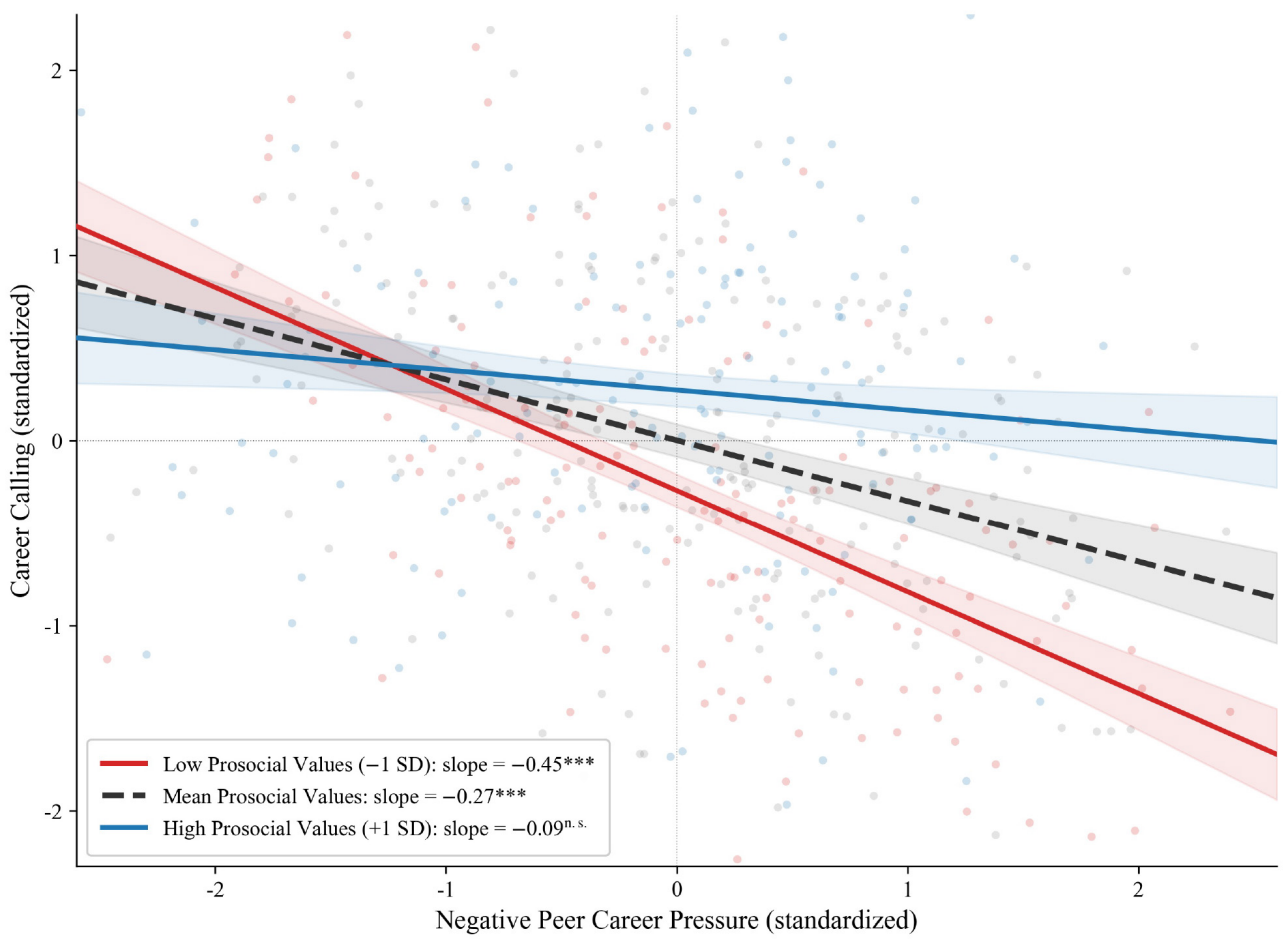
Simple slopes of negative peer career pressure on career calling at three levels of internalized prosocial values (*N* = 524).

**Figure 5:**
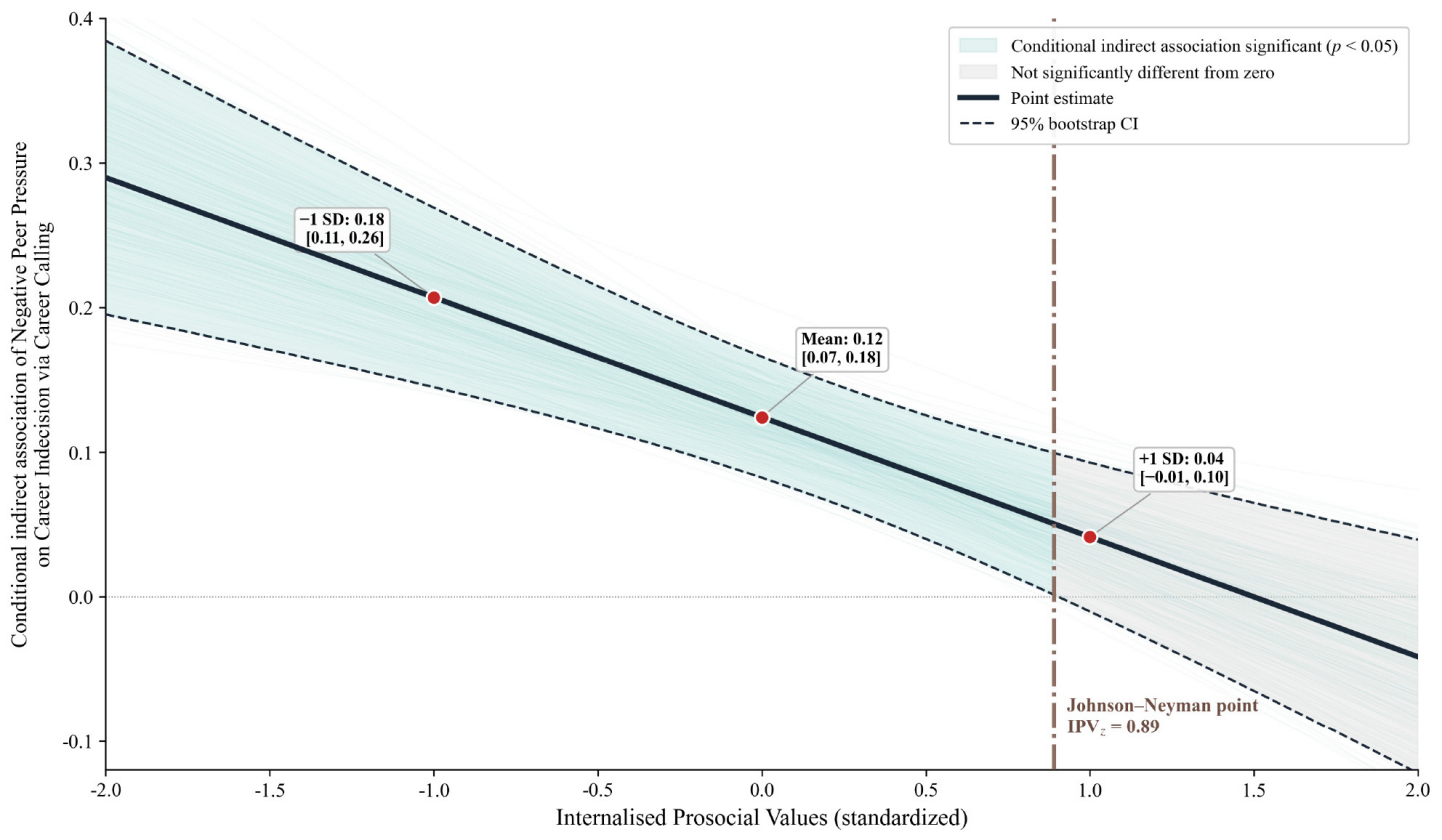
Conditional indirect association of negative peer career pressure with career indecision via career calling across the range of internalized prosocial values. The shaded region represents the 95% confidence band. The Johnson–Neyman transition point indicates the level of internalized prosocial values at which the conditional indirect association becomes non-significant.

The index of moderated mediation for the negative peer pressure to career indecision pathway was −0.08 (95% bootstrap CI [−0.13, −0.04]), supporting H5. The simple slopes of negative peer career pressure on career calling at three levels of internalized prosocial values are plotted in Figure 4. The negative sign of the index indicates that the (positive) indirect association of negative peer pressure with career indecision through career calling decreases as prosocial value internalization rises. The conditional indirect associations at three levels of internalized prosocial values are reported in Table 6. At low internalization (−1 SD), the indirect association of negative peer pressure with career indecision via calling was substantial (0.18, 95% CI [0.11, 0.26]). At the mean, the association was attenuated (0.12, 95% CI [0.07, 0.18]). At high internalization (+1 SD), the association was further reduced and no longer statistically distinguishable from zero (0.04, 95% CI [−0.01, 0.10]). The parallel index for the positive peer support pathway was not significant (−0.03, 95% CI [−0.06, 0.01]), consistent with the asymmetric moderation prediction. Figure 5 displays the conditional indirect association across the full range of internalized prosocial values, with the Johnson–Neyman transition point indicating where the association becomes non-significant.

**Table 6:** Conditional indirect associations: negative peer pressure on career indecision via calling.

Prosocial values level	Indirect est.	Boot SE	95% bootstrap CI	Significant
Low (−1 SD)	0.18	0.04	[0.11, 0.26]	Yes
Mean	0.12	0.03	[0.07, 0.18]	Yes
High (+1 SD)	0.04	0.03	[−0.01, 0.10]	No
Index of moderated mediation	−0.08	0.02	[−0.13, −0.04]	Yes

*N* = 524. Bootstrap 5,000 resamples. LMS estimation.

The index of moderated mediation equals the product of the interaction coefficient and the calling-to-indecision path: (+0.18) × (−0.46)≈−0.08.

### 4.6 Robustness checks

Several robustness checks were conducted. The structural model was re-estimated with each control variable removed in turn; the substantive conclusions did not change. The moderated mediation was re-estimated separately by gender, year of study, and discipline; the interaction coefficient remained positive and statistically significant in all subgroups, with magnitudes ranging from 0.13 to 0.22, supporting cross-subgroup robustness of the buffering pattern. An alternative specification with internalized prosocial values as a mediator rather than a moderator yielded inferior fit (Δχ^2^ = 27.4, Δ*df* = 3, *p* < 0.001), supporting the moderation specification adopted in the main analysis.

To evaluate the plausibility of the proposed directional specification, three alternative structural models were tested. Alternative Model A reversed the calling-to-indecision path (i.e., career indecision → career calling); this model showed inferior fit (ΔAIC = +31.6, ΔBIC = +31.6). Alternative Model B specified career indecision as a predictor of perceived peer pressure (reversing the peer pressure → indecision path); this model also showed inferior fit (ΔAIC = +18.9, ΔBIC = +18.9). Alternative Model C specified a reciprocal relationship between career calling and career indecision using an instrumental-variable approach; the reciprocal path from indecision to calling was not significant (β = −0.07, *p* = 0.231), whereas the calling-to-indecision path remained significant (β = −0.41, *p* < 0.001). Although these comparisons do not establish causality (which requires longitudinal data), they are consistent with the directional specification proposed in the present model.

## 5 Discussion

### 5.1 Asymmetric peer influence on career indecision

The findings provide partial support for H1a and full support for H1b, indicating that positive peer career support and negative peer career pressure are asymmetrically associated with career indecision. Negative peer pressure was directly associated with higher career indecision, whereas the association of positive peer support was entirely transmitted through the meaning-based pathway. This asymmetry echoes earlier work suggesting that not all forms of peer involvement contribute equally to career development (Felsman and Blustein, 1999; Cheung and Arnold, 2014; Schellenberg et al., 2022), and it extends recent contemporary career orientation research by demonstrating that the differentiation between supportive and pressuring peer dynamics is empirically necessary in the Chinese context (Hirschi and Koen, 2021; Akkermans and Hirschi, 2023). The direct association of pressure with indecision suggests that beyond the meaning-based route, pressure may introduce decisional noise through additional channels, plausibly including upward social comparison anxiety (Xu and Li, 2024), increased cognitive load from monitoring peer trajectories (Wen and Jin, 2025), and externally referenced self-evaluation that bypasses the meaning-based system (Shang and Bao, 2025). Practically, the asymmetry implies that supporting students by amplifying positive peer dynamics alone is insufficient; explicit attention to dampening negative peer pressure is also required (Zhou et al., 2024; Marcionetti and Zammitti, 2025).

The cultural context enriches the interpretation of this asymmetry. In Hofstede's framework, Chinese society scores high on collectivism, meaning that in-group norms and expectations exert strong pressure on individual decision making (Hofstede, 2001). Triandis's vertical-collectivism construct suggests that peer career pressure in Chinese universities carries a normative force that may be less prominent in individualist cultural contexts (Triandis, 1995). The interdependent self-construal that predominates in such contexts (Singelis, 1994) means that negative peer evaluations are experienced not merely as external noise but as threats to relational identity, which could explain why peer pressure shows a direct association with indecision that is not fully mediated through the meaning-based pathway. Kağıtçıbaşı's model of the autonomous-relational self (Kağıtçıbaşı, 2007) further suggests that Chinese university students, who are navigating between growing personal autonomy and deep relational embeddedness, are particularly vulnerable to the dissonance created by peer career pressure. Future cross-cultural research should test whether the asymmetric pattern replicates in individualist cultural contexts where the normative weight of peer comparison may differ.

It is also instructive to compare the present result with international evidence on peer associations in career development. Studies of European and North American samples have generally found that supportive peer relationships are associated with career exploration and decreased indecision, but recent qualitative work emphasizes that peer dynamics are often experienced as ambivalent rather than uniformly supportive (Felaco et al., 2023; Parola et al., 2023). The present finding that supportive and pressuring peer dynamics co-exist within the same friendship networks, and show opposing associations with the same outcome, suggests that the ambivalent character of peer influence is not specific to the Chinese context, even though dormitory cohabitation and dense class-based group life may amplify its salience.

### 5.2 Career calling as a differentiating mechanism

Consistent with H2a, H2b, H3, H4a, and H4b, career calling mediated both peer pathways, but with theoretically interesting differences. Full mediation of positive peer support and partial mediation of negative peer pressure suggest that the meaning-based system is the dominant route through which supportive peers are linked to reduced indecision, whereas pressuring peers also appear to operate through additional non-meaning-based routes. This pattern aligns with the broader career construction literature, which emphasizes meaning making as the integrative core of career development (Savickas, 2013; Hirschi and Pang, 2023), and with the calling literature's claim that calling functions as an internal compass that organizes decisions and reduces ambiguity (Duffy et al., 2018; Praskova et al., 2015; Beloborodova and Leontiev, 2024). The differentiated mediation pattern is also consistent with recent Chinese-context work showing that calling is positively associated with career commitment, learning engagement, and career adaptability among undergraduates (Chen and Zhang, 2023; Chen et al., 2024; Zhang et al., 2022), with longitudinal evidence that calling and authentic vocational identity reciprocally reinforce one another over time (Zhang et al., 2017; Jia et al., 2021), and with the broader finding that professional identity is associated with greater career adaptability through deeper learning engagement in Chinese engineering samples (Liu et al., 2023a).

The choice of career calling over self-efficacy as the focal mediator merits comment. SCCT has traditionally emphasized self-efficacy and outcome expectations as the primary cognitive-person variables linking contextual inputs to career outcomes (Lent et al., 1994). The present findings do not challenge the importance of these constructs; rather, they suggest that meaning-based constructs such as calling represent a complementary pathway that captures a different facet of the peer-to-indecision link. Whereas self-efficacy captures whether students feel capable of making career decisions, calling captures whether they have a coherent sense of direction and purpose that organizes the decision-making process (Hirschi and Pang, 2023). The partial mediation finding for negative peer pressure further implies that additional mediators, including self-efficacy and outcome expectations, likely account for the residual direct association, pointing toward a multi-mediator model as a productive direction for future research.

The differentiated mediation pattern also has practical implications. Interventions that aim to enrich the meaning-based system, for example by helping students articulate their vocational identity narratives, structured calling discernment exercises, and reflection on long-term contribution goals (Beloborodova and Leontiev, 2024; Hirschi and Pang, 2023), are likely to fully translate gains from positive peers but only partially shield students from the indecision-generating associations of negative peers. Complementary interventions targeting the non-meaning-based routes, such as social comparison reappraisal, peer normative interventions, and decisional load reduction (Xu and Li, 2024; Wen and Jin, 2025; Zhou et al., 2024), are therefore needed to complete the protective architecture.

### 5.3 Internalized prosocial values as a contextual buffer

The most novel finding of the study is the buffering moderation of internalized prosocial values on the negative peer pressure to calling pathway. Consistent with H5, the negative association of pressure with calling was substantially attenuated at high levels of value internalization and non-significantly different from zero at the upper level. The asymmetry of the moderation, namely the absence of a parallel interaction on the positive peer pathway, supports the integration mechanism: internalized prosocial values and positive peer support appear to operate through overlapping integration pathways and therefore yield additive rather than interactive associations, whereas internalized prosocial values provide a non-peer-referenced source of meaning that specifically counteracts externally referenced pressure. This pattern is consistent with self-determination theory's account of how internalized values support autonomous motivation under contextual challenge (Ryan and Deci, 2017; Hirschi, 2020; Vansteenkiste et al., 2020), and with recent Chinese-context evidence linking prosocial value integration to value identity, prosocial behavior, and psychological adjustment (Xue et al., 2024; Zhao, 2022; Tong, 2025).

The distinction between institutional curriculum exposure and individual value internalization is important for interpreting this finding. The moderator in the present model captures the degree to which students have personally integrated prosocial and civic commitments into their value systems, not the amount of curricular exposure they have received. This distinction matters because exposure without internalization (what self-determination theory terms external or introjected regulation) would not be expected to attenuate the negative association of peer pressure with calling; only integrated values provide the stable, autonomous motivational base that can counteract externally referenced comparison (Ryan and Deci, 2017; Vansteenkiste et al., 2020). The implication for universities is that mere delivery of values-education content is insufficient; pedagogical approaches that support genuine value internalization through autonomy support, perspective-taking, and experiential engagement are needed (Tong, 2025; Tian, 2025).

It is also worth noting how internalized prosocial values relate to collectivist cultural orientations. In Hofstede's framework, collectivist cultures emphasize group loyalty and shared obligations (Hofstede, 2001). Internalized prosocial values can be understood as a specific instance of value integration that aligns individual agency with prosocial commitments, thereby reducing the tension between personal career aspirations and group-referenced expectations. In Kağıtçıbaşı's terms (Kağıtçıbaşı, 2007), prosocial value internalization helps resolve the autonomy-relatedness dialectic by providing a framework within which personal career choices and social contribution are experienced as compatible rather than conflicting. This culturally informed interpretation suggests that the buffering mechanism may be particularly potent in collectivist contexts but should also operate, perhaps with reduced magnitude, in individualist contexts where prosocial commitments similarly provide a stable non-competitive reference point for career meaning.

This finding has clear theoretical implications. It positions internalized prosocial values not as an additive contextual support but as a specific buffer against the maladaptive class of contextual stressors, thereby refining the Social Cognitive Career Theory account of contextual barriers (Lent and Brown, 2019; Lent et al., 1994; Sheu et al., 2018). The buffering pattern is also conceptually consonant with the Psychology of Working framework, which highlights how meaning-based, prosocially oriented work resources can offset contextual constraints (Duffy et al., 2016). Because the moderator captures individual value internalization rather than curriculum-specific content, the buffering mechanism is, in principle, portable to other national contexts in which civic, character, or service-learning curricula promote prosocial commitments. Future cross-cultural research should test whether comparable internalization processes produce analogous buffering patterns in contexts with different cultural orientations on the collectivism-individualism dimension (Hofstede, 2001; Triandis, 1995).

### 5.4 Practical implications

The results suggest several practical implications for Chinese higher education governance and for international comparators, although these implications should be interpreted with caution given the cross-sectional design. The asymmetric peer associations imply that peer-aware career education should be designed bidirectionally: structured peer learning communities can amplify positive peer support (Schellenberg et al., 2022; Wang et al., 2025b), while normative interventions and reflective practice can dampen the negative association of upward social comparison (Xu and Li, 2024; Wen and Jin, 2025). Career calling cultivation should be elevated from a peripheral concern to a central component of career education curricula, because it functions as the dominant translation mechanism through which peer experiences may be linked to decision-making clarity (Duffy et al., 2018; Chen et al., 2024; Beloborodova and Leontiev, 2024). Programmes that support genuine internalization of prosocial values, rather than surface-level compliance with curricular requirements, should be integrated with career education, because internalized prosocial values appear to function as a specific psychological resource that attenuates the maladaptive association of peer pressure with career meaning (Xue et al., 2024; Tong, 2025). Pedagogical strategies that promote autonomy-supportive engagement with civic and ethical content, including service-learning, perspective-taking exercises, and reflective journaling, may be particularly effective in fostering value internalization (Ryan and Deci, 2017; Tian, 2025). The compatibility of prosocial value cultivation with career calling cultivation is theoretically expected and empirically supported (Hirschi and Pang, 2023; Zhang et al., 2015), and the operational integration of these two pillars is a feasible and policy-relevant step for university administrators (Yang et al., 2024; Wang et al., 2023).

## 6 Conclusions, limitations, and future directions

This study tested a moderated mediation model in which positive peer career support and negative peer career pressure jointly are associated with career indecision through career calling, with internalized prosocial values attenuating the negative association of peer pressure with calling. Drawing on a survey of 524 Chinese undergraduates and a combination of confirmatory factor analysis, latent moderated structural equations, and bias-corrected bootstrap procedures, three findings emerged. Peer influence is asymmetrically associated with career indecision, with pressure contributing directly and support contributing indirectly. Career calling differentially mediates the two peer pathways, fully transmitting positive support and partially transmitting negative pressure. Internalized prosocial values attenuate the negative pressure pathway, reducing the conditional indirect association to non-significance at high levels of value internalization. Together, these findings extend the Social Cognitive Career Theory by identifying a psychologically targetable contextual buffer, and they offer provisional guidance for designing peer-aware and values-centered career education in Chinese universities, situated within the collectivist cultural context that amplifies peer salience (Hofstede, 2001; Triandis, 1995).

Several limitations should be acknowledged. Most importantly, the cross-sectional design precludes strong causal inference, and the directional terminology used in the structural model (e.g., paths from peer influence to calling to indecision) reflects the theoretical specification rather than empirically established causation. Although temporal separation of measures within the survey and multiple common-method-bias safeguards (Harman's test, CLF analysis, marker-variable adjustment) reduce common method concerns, a multi-wave longitudinal design that tracks the dynamic interplay between peer experiences, calling, and indecision across the undergraduate years would substantially strengthen the evidence base (Zhang et al., 2017; Jia et al., 2021). Such a design would also permit the use of cross-lagged panel models or latent growth curve models to test the temporal ordering that the present cross-sectional model assumes but cannot confirm. Future studies might also incorporate dyadic and network measures of peer influence rather than the self-reported peer perception measures used here, which would allow estimation of objective peer composition associations in addition to perceived peer climate (Schellenberg et al., 2022; Felaco et al., 2023).

The single-institution sample, while large and balanced, limits generalizability across Chinese institutional types. Replication in vocational and elite institutions, and in eastern, central, and western regions, is needed to establish broader generalizability (Gao et al., 2025; Wang et al., 2023). The internalized prosocial values scale, although psychometrically adequate and conceptually grounded in self-determination theory, was developed for the present study; external replication and further validation in other Chinese institutions, and in cross-national samples with comparable civic or character education curricula, is needed (Tong, 2025; Zhang and Fagan, 2016). In particular, future validation work should include test-retest reliability, criterion-related validity against behavioral indicators of prosocial commitment, and cross-cultural measurement invariance testing.

The model focused on a single mediator (career calling) and a single moderator (internalized prosocial values). Future studies should consider additional mediators such as career adaptability, decision-making self-efficacy, outcome expectations, and proactive personality (Xu et al., 2025; Liu et al., 2026; Rudolph et al., 2017; Akkermans and Hirschi, 2023), and additional moderators such as parental career-related support, institutional career advising structures, and the structure of the local peer network (Parola and Marcionetti, 2022; Yang et al., 2024; Wang et al., 2024). The mediation and moderation patterns documented here are likely to be embedded in a richer relational and motivational ecology that future work can unpack. Recent work on basic psychological needs satisfaction (Xu et al., 2023), growth mindset (Liu et al., 2026), and group attachment (Wang et al., 2025b) suggests promising candidate constructs for such an extension.

Despite these limitations, the present study makes a clear contribution to the literature on contextual factors in undergraduate career development by establishing that peer associations with career indecision are bidirectional, that they are linked to indecision primarily through a meaning-based mechanism captured by career calling, and that internalized prosocial values function as a specific buffer against the maladaptive class of peer dynamics. These findings reposition prosocial value internalization as an active component of the career development ecology and offer a portable framework for international comparators interested in fostering civic or character value internalization through education. They also reinforce the case for peer-aware, meaning-oriented, and values-centered career support in Chinese higher education and beyond.

## Data Availability

The raw data supporting the conclusions of this article will be made available by the authors, without undue reservation.
